# Activin-A is up-regulated in severe asthma, attenuates allergic responses and is associated with angiogenesis

**DOI:** 10.1186/2045-7022-3-S1-O4

**Published:** 2013-05-03

**Authors:** Konstantinos Samitas, Sofia Tousa, Nikolaos Poulos, Eleftherios Zervas, Maria Semitekolou, Catherine M Hawrylowicz, Harsha Kariyawasam, Georgina Xanthou, Mina Gaga

**Affiliations:** 1 7th Resp Dept and Asthma Centre, Athens Chest Hospital, Greece; 2 Biomedical Research Foundation of the Academy of Athens, Cellular Immunology Laboratory, Greece; 3 Kings College London, Guys Hospital, UK; 4 Dept of Allergy and Medical Rhinology, Royal National Throat Nose Ear Hospital, UCL, UK

## Background

Activin-A (Act-A) is a pleiotropic cytokine belonging to the TGF-β superfamily. Recent studies from our group have shown that Act-A suppresses mouse allergic responses; however its effects on human asthma remain unknown.

## Objectives

Determine Act-A expression in healthy controls (CTRL) and asthmatics with mild-moderate (MMA) and severe asthma (SA), identify its cellular sources, examine its signaling mediators' expression and correlations with disease severity and airway remodeling, and investigate its effects on allergic inflammatory responses.

## Methods

Act-A expression was quantified in the serum, BALF (by ELISA) and bronchial tissue samples (IHC) obtained from CTRL (n=41), MMA (n=46) and SA (n=26). Act-A signaling expression (ActRIIA, ALK4, pSMad2/3) and remodeling markers (basement membrane thickness, goblet cell hyperplasia and angiogenesis) were also assessed in bronchial tissue (IHC/IF/Confocal). Moreover, naive T cells from atopic CTRLs and MMA/SA were isolated and cultured *ex vivo* alone or in the presence of allergen and/or rAct-A and/or dexamethasone and further utilized in co-cultures with naive T cells and in adoptive transfer experiments using NOD/SCID mice in a humanized model of experimental asthma.

## Results

Act-A levels were significantly increased in the serum, BALF and bronchial tissue of asthmatics, especially in the subepithelium in SA. Act-A was expressed by T cells, neutrophils, mast cells, macrophages and endothelial cells. ActRIIA, ALK4 and pSMad2/3 expression was downregulated especially in SA. Regarding remodeling, subepithelial Act-A expression correlated with tissue angiogenesis and Act-A/ALK4 were co-expressed in endothelial cells pointing to active signaling. Act-A attenuated allergic responses of human naive T cells (decreased IL4, IL5, IL13) of atopic CTRL and asthmatics through the induction of IL-10 producing regulatory-like T cells (Act-A-iTregs) and enhanced dexamethasone-induced T cell suppression. Adoptive transfer of Act-A-iTregs in the NOD/SCID mouse model suppressed experimental asthma.

## Conclusions

Act-A expression is increased in severe asthma however its signaling pathways is downregulated. Act-A suppresses airway inflammation *ex vivo* through the induction of functional Act-A-iTregs. Our data suggest that Act-A plays a crucial role in the inflammatory and angiogenetic processes in asthma. Ongoing *in vitro* studies will further elucidate its specific role in angiogenesis.

